# Accelerating Postoperative Recovery: The Impact of Early Mobility Protocols in Surgical Intensive Care Unit (SICU) Patients After Open Cholecystectomy

**DOI:** 10.7759/cureus.80692

**Published:** 2025-03-16

**Authors:** Binu Xavier, Sasi Vaithilingan, Latha R

**Affiliations:** 1 Medical Surgical Nursing, Vinayaka Mission's Research Foundation, Deemed to be University, Salem, IND; 2 Child Health Nursing, Vinayaka Mission's College of Nursing, Puducherry, IND; 3 Microbiology, Aarupadai Veedu Medical College and Hospital, Puducherry, IND

**Keywords:** cholecystectomy, early mobility protocol, functional independence, postoperative recovery, sicu

## Abstract

Background

Diseases of the biliary system and gallbladder pose a significant global public health burden, affecting millions annually. Conditions such as gallstone formation, cholecystitis, and biliary dysfunction contribute to gastrointestinal morbidity, often necessitating surgical intervention. Gallstone prevalence ranges between 10% and 15%, with post-surgical recovery in open cholecystectomy patients being hindered by limited mobility, leading to prolonged hospital stays. This study investigates the impact of implementing an early mobility protocol on postoperative recovery in patients who have undergone open cholecystectomy.

Methods

This study included a total of 24 patients who underwent open cholecystectomy, who were randomly assigned to two groups, with 12 participants in each. Baseline demographic and clinical characteristics, including age, gender, education level, religion, body mass index (BMI), and preoperative pain scores, were systematically documented to ensure comparability between the groups. The experimental group followed a structured early mobility protocol, consisting of supervised mobility exercises such as sitting, standing, breathing exercises, abdominal exercises, and short-distance ambulation, conducted twice daily from the first postoperative day until the fourth day. In contrast, the control group received standard postoperative nursing care without additional mobility interventions. Recovery outcomes, including mobility levels, functional independence, and patient satisfaction, were evaluated using validated assessment scales. Statistical analyses were performed using SPSS version 28.0 (IBM Corp., Armonk, NY, USA).

Results

Prior to the intervention, all patients exhibited complete dependence on mobility. Following the intervention, 83.3% of patients in the experimental group attained moderate mobility, with 58.3% achieving partial functional independence by the fourth postoperative day (p < 0.001). The experimental group demonstrated a mean improvement in mobility scores of 4.67, significantly higher than the 0.24 mean improvement in the control group (p < 0.001). Functional independence scores in the experimental group increased by a mean difference of 19.08 (p < 0.001). A strong positive correlation was observed between mobility and functional independence (r = 0.744, p < 0.01), reinforcing the efficacy of early mobilization.

Conclusion

The implementation of an early mobility protocol significantly improved mobility, functional independence, and patient satisfaction in open cholecystectomy patients, demonstrating its importance in postoperative care. The protocol’s effectiveness was consistent across different demographic variables, suggesting broad applicability. Larger studies are recommended to further validate these findings.

## Introduction

Gallbladder and biliary diseases (GABDs) are prevalent digestive disorders, including gallstones (cholelithiasis), gallbladder inflammation (cholecystitis), cholangitis, and bile duct blockages [1]. Over 20 million adults in the United States are affected by gallbladder disease, with an estimated annual healthcare cost of $6.2 billion. Gallstone disease (GSD) is a significant global health concern, affecting 10-15% of adults in Western nations [2]. While most cases remain asymptomatic, approximately 1-2% of patients develop symptoms annually, necessitating surgical intervention, making cholecystectomy one of the most frequently conducted surgical procedures across the globe [3].

The prevalence of gallstones varies across populations, ranging from 10% to 15% globally, and approximately 20% of affected individuals experience biliary colic [4]. If untreated, gallstones carry a 1-2% annual risk of complications, which may lead to severe conditions such as systemic inflammatory response syndrome and sepsis [5]. Gallbladder diseases significantly impact long-term quality of life, leading to frequent hospitalizations and surgical procedures, thereby imposing a substantial financial burden [6]. The overall healthcare expenditure associated with cholelithiasis and cholecystitis in the U.S. increased from $2.2 billion in 2009 to $4 billion by 2014 [7].

In India, the prevalence of gallstones is estimated at 3-5%, with North Indians being seven times more susceptible than South Indians [5]. Women are affected more frequently than men, with a prevalence ratio of 2.8:1. Laparoscopic cholecystectomy is currently the preferred and standard approach for treating gallstones, though the conversion rate to open surgery varies between 0-20%. Open cholecystectomy requires a longer recovery period, leading researchers to explore strategies to enhance postoperative recovery [8]. Early mobilization has emerged as a key approach to accelerating recovery, reducing complications, and improving patient outcomes following surgery. This study aims to evaluate the effectiveness of early mobilization in enhancing postoperative recovery following open cholecystectomy, assess improvements in mobility levels and functional independence among surgical intensive care unit (SICU) patients, and determine the impact of early mobility on patient satisfaction and overall clinical outcomes.

## Materials and methods

Study design and setting

An experimental study was conducted from November 2022 to January 2024 in the SICU of IQ City Medical College Hospital, West Bengal. Ethical approval was obtained from the Institutional Ethics Committee of IQ City Medical College Hospital (Approval No.: IQMC/IEC-10/LTR/10(15)/53).

Participants and sampling

A total of 24 patients who had undergone open cholecystectomy were recruited for the study using a simple random sampling technique. To ensure uniformity in postoperative conditions, only patients who were within 24 hours post-surgery, conscious, and able to communicate were included in the study. Additionally, patients experiencing mild to moderate pain were selected to assess the effectiveness of the early mobility intervention within this specific pain range. These patients were randomly assigned to either the experimental group (n = 12) or the control group (n = 12).

Inclusion criteria

Only patients who were within 24 hours post-surgery were included in the study. This ensured that the patients were in the early postoperative phase, where pain management was crucial for recovery. Patients who were conscious and able to communicate were selected, as this was essential to accurately assess and monitor their pain levels and overall condition during the study. Additionally, the study focused on patients experiencing mild to moderate pain, as this criterion was important to evaluate the effectiveness of pain management interventions within this specific pain range.

Exclusion criteria

Patients were excluded from the study if they met any of the following conditions: individuals with fractures were not included due to the complexities associated with pain management, which could impact the study's outcomes. Similarly, patients with bleeding disorders were excluded as these conditions could pose additional risks during postoperative recovery and pain management. Additionally, patients with orthostatic hypotension were not considered for the study due to the potential risk of complications arising from blood pressure fluctuations during postoperative care.

Intervention

The experimental group received an early mobility protocol twice daily until the fourth postoperative day. The protocol included structured exercises lasting 20-25 minutes per session, comprising sitting, standing, breathing exercises, abdominal exercises, and walking. The control group was provided with standard nursing care without additional mobility interventions.

Data collection tools and measures

Data collection for this study was conducted using a comprehensive, validated tool designed to ensure accuracy and reliability in assessing patient outcomes. This tool consisted of three distinct sections, each targeting a specific aspect of the study. The first section, Part A, collected demographic and baseline characteristics, including details such as age, gender, education level, religion, pain score, and BMI. These variables were recorded to ensure comparability between the experimental and control groups and to assess potential confounding factors.

The second section, Part B, focused on evaluating patient mobility using the modified Johns Hopkins Highest Level of Mobility (JH-HLM) scale. This scale assessed patients' ability to perform various movements, including repositioning in bed, transitioning from lying to sitting, moving to a chair, standing, and walking independently. The JH-HLM scale is widely recognized for its effectiveness in assessing mobility in clinical settings, making it a reliable tool for measuring the impact of early mobility interventions [9]. The mobility levels were categorized into dependent, moderately dependent, and independent statuses, providing a structured way to analyze improvements over time.

The third section, Part C, measured patient satisfaction using a structured 10-point rating scale, ranging from 1 (completely dissatisfied) to 10 (highly satisfied). This scale captured patients' perceptions of their postoperative recovery experience, including their comfort levels, perceived ease of movement, and overall satisfaction with the care received.

To ensure the validity and reliability of the data collection tools, a rigorous validation process was conducted by a panel of medical and nursing professionals specializing in postoperative care and mobility assessment. The reliability of the tools was confirmed through Cronbach’s alpha analysis, yielding a value of 0.91, which indicates a high level of internal consistency and reliability [10]. This validation process ensured that the study measurements were both statistically robust and clinically relevant, enhancing the credibility of the findings.

Classification of outcomes

The outcomes of the study were classified based on the mobility levels of the patients, as assessed using a standardized scale. This classification was divided into three categories: patients who scored between 1 and 3 were considered dependent, indicating that they required a high level of assistance for mobility tasks. Patients with scores ranging from 4 to 6 were categorized as moderately dependent, meaning they needed a moderate amount of assistance for mobility. Finally, patients who achieved scores between 7 and 8 were classified as independent, signifying that they were capable of performing mobility tasks without external assistance.

Functional independence levels

The study further classified patients' functional independence into three distinct categories. Patients classified as independent were able to perform daily tasks without any external assistance. Those categorized under modified dependence required some level of support or assistance for certain tasks but retained partial independence. Lastly, patients classified as completely dependent required full assistance for performing functional tasks, indicating a total reliance on caregivers for mobility and daily activities.

Patient satisfaction

Patient satisfaction was evaluated using a 5-point scale, which ranged from "not at all satisfied" to "very much satisfied." This scale provided a structured approach to measure the patients' overall satisfaction with the care and treatment they received during the study, capturing varying levels of contentment and identifying areas for potential improvement in patient care.

Statistical analysis

Descriptive and inferential statistical analyses were employed to evaluate the effectiveness of the early mobilization protocol (EMP) in postoperative recovery. Descriptive statistics, including mean, standard deviation, frequency, and percentage distributions, were used to summarize demographic variables, baseline characteristics, and outcome measures. Inferential statistics involved the application of the chi-square test (χ²) to compare categorical variables such as mobility levels, functional independence, and patient satisfaction between the experimental and control groups. Paired t-tests were conducted to analyze within-group differences in mobility and functional independence before and after the intervention, while independent t-tests were used to compare between-group differences. To assess the relationship between post-test mobility scores and functional independence, Pearson’s correlation analysis was performed, with a strong positive correlation indicating that improved mobility significantly contributed to enhanced functional independence. Statistical significance for all tests was set at p < 0.05, ensuring standard confidence levels, and all analyses were carried out using SPSS software (version 28.0, IBM Corp., Armonk, NY, USA) for accuracy and reliability.

## Results

Demographic and baseline characteristics

The demographic and baseline characteristics of patients in both the experimental and control groups are outlined in Table 1. The results of the chi-square test indicated that there were no statistically significant differences between the groups in terms of age (p = 0.306), gender (p = 1.000), BMI (p = 0.871), education level (p = 0.938), religion (p = 0.875), and pain score distribution. These findings suggest that both groups were comparable and well-matched at the beginning of the study.

**Table 1:** Demographic and baseline characteristics BMI: Body Mass Index A p-value of p<0.05 was considered statistically significant. Not significant values are denoted with n.s. (n.s.: not significant)

Demographic Variables	Experimental Group (n=12) N (%)	Control Group (n=12) N (%)	Chi-Square Test (χ²)	p-Value
Age (years)				
31 – 40	2 (16.7%)	2 (16.7%)	3.619	0.306^n.s^
41 – 50	8 (66.6%)	4 (33.3%)		
51 – 60	2 (16.7%)	5 (41.7%)		
>60	0 (0%)	1 (8.3%)		
Gender				
Male	4 (33.3%)	4 (33.3%)	0.000	1.000^n.s^
Female	8 (66.7%)	8 (66.7%)		
BMI				
18.5 – 24.9	6 (50%)	7 (58.3%)	0.277	0.871^n.s^
25 – 29.9	3 (25%)	2 (16.7%)		
30 – 34.9	3 (25%)	3 (25%)		
Education				
High school	7 (58.4%)	6 (50%)	0.410	0.938^n.s^
Primary school	1 (8.3%)	2 (16.7%)		
Graduates	3 (25%)	3 (25%)		
Postgraduates & above	1 (8.3%)	1 (8.3%)		
Religion				
Hindu	7 (58.3%)	8 (66.6%)	0.267	0.875^n.s^
Muslim	3 (25%)	2 (16.7%)		
Christian	2 (16.7%)	2 (16.7%)		
Pain Score				
4 – 6	12 (100%)	12 (100%)	---	---

Effect of early mobility protocol on mobility levels

The study commenced on postoperative day one (24 hours after surgery) to ensure patient stability before initiating the mobility protocol. As illustrated in Table 2, the pre-intervention mobility levels were identical for both groups, with all patients categorized as having dependent mobility (100%). After the intervention, a notable improvement was observed in the experimental group, with 83.3% of participants progressing to moderately dependent mobility and 16.7% attaining independent mobility. Conversely, all individuals in the control group continued to exhibit fully dependent mobility. The results of the chi-square test indicated a statistically significant difference between the two groups (p = 0.0001), underscoring the effectiveness of the intervention in enhancing mobility levels.

**Table 2:** Level of mobility pre- and post-intervention A p-value of p < 0.0001 was considered statistically significant. Significant p-values are denoted with an asterisk (p<0.0001)

Time	Level of Mobility	Scale Score	Experimental Group (n=12)	Control Group (n=12)	Chi-Square Test (χ²)	p-value
Pre	Dependent mobility	1 – 3	12 (100%)	12 (100%)	---	---
Moderately dependent mobility	4 – 6	0	0	---		
Independent mobility	7 – 8	0	0	---		
Post	Dependent mobility	1 – 3	0	12 (100%)	24.0	0.0001*
Moderately dependent mobility	4 – 6	10 (83.3%)	0	---		
Independent mobility	7 – 8	2 (16.7%)	0	---		

Functional independence outcomes

Table 3 illustrates the functional independence levels before and after the intervention. Pre-intervention, all patients in both groups were classified as having modified dependence (100%). Following the intervention, 58.3% of the experimental group achieved independent functional status, while 41.7% remained at modified dependence. The control group, however, exhibited no improvement, with all patients retaining their pre-intervention functional dependence levels. The chi-square test (χ² = 9.882, p = 0.003) indicates a statistically significant enhancement in functional independence within the experimental group.

**Table 3:** Level of functional independence pre- and post-intervention A p-value of p<0.05 was considered statistically significant. Significant p-values are denoted with an asterisk (p<0.05)

Time	Functional Independence Level	Scale Score	Experimental Group (n=12)	Control Group (n=12)	Chi-Square Test (χ²)	p-value
Pre	Complete Dependence	18 – 42	0	0	---	---
Modified Dependence	43 – 85	12 (100%)	12 (100%)	---		
Independent	86 – 126	0	0	---		
Post	Complete Dependence	18 – 42	0	0	9.882	0.003*
Modified Dependence	43 – 85	5 (41.7%)	12 (100%)	---		
Independent	86 – 126	7 (58.3%)	0	---		

Effectiveness of early mobility protocol on mobility and functional independence

Table 4 presents the statistical comparison of pre- and post-test mobility scores. The mean mobility score in the experimental group significantly increased from 1.33 to 6.00 (mean difference = 4.67, t = 20.765, p = 0.0001), whereas the control group showed a minimal, non-significant change from 1.41 to 1.67 (mean difference = 0.24, t = 1.393, p = 0.191). The results confirm the effectiveness of the early mobility protocol in improving patient mobility.

**Table 4:** Effectiveness of early mobility protocol on highest mobility A p-value of p<0.0001 was considered statistically significant. Significant p-values are denoted with an asterisk (p<0.0001)

Group	Pretest (Mean ± SD)	Post-Test (Mean ± SD)	Mean Difference	Paired t-test	p-value
Experimental Group	1.33 ± 0.49	6.00 ± 0.60	4.67	20.765	0.0001*
Control Group	1.41 ± 0.66	1.67 ± 0.77	0.24	1.393	0.191

Similarly, Table 5 reports the functional independence scores. The experimental group experienced a significant improvement in functional independence, with mean scores increasing from 64.83 to 83.91 (mean difference = 19.08, t = 16.301, p = 0.0001). In contrast, the control group showed negligible improvement (60.75 to 60.91, mean difference = 0.16, t = 1.483, p = 0.166), demonstrating the superiority of the intervention in enhancing functional independence.

**Table 5:** Effectiveness of early mobility protocol on functional independence A p-value of p<0.0001 was considered statistically significant. Significant p-values are denoted with an asterisk (p<0.0001)

Group	Pretest (Mean ± SD)	Post-Test (Mean ± SD)	Mean Difference	Paired t-test	p-value
Experimental Group	64.83 ± 6.58	83.91 ± 7.90	19.08	16.301	0.0001*
Control Group	60.75 ± 5.97	60.91 ± 6.08	0.16	1.483	0.166

Correlation between mobility and functional independence

Table 6 presents the correlation analysis between post-test mobility and functional independence scores. A strong positive correlation was observed in the experimental group (r = 0.744, p = 0.006), suggesting that improvements in mobility significantly enhanced functional independence. In contrast, the control group exhibited a weak and non-significant correlation (r = -0.051, p = 0.875), indicating no meaningful relationship between these variables.

**Table 6:** Correlation between post-test mobility and functional independence A p-value of p<0.05 was considered statistically significant. Not significant values were denoted as n.s (n.s.: Not significant)

Group	Variable	Mean ± SD	Pearson's Correlation (r)	p-value
Experimental Group	Highest Mobility	6.0 ± 0.60	0.744	0.006^n.s^
	Functional Independence	83.91 ± 7.90		
Control Group	Highest Mobility	3.91 ± 0.79	-0.051	0.875
	Functional Independence	66.66 ± 5.24		

Patient satisfaction

As shown in Table 7, the experimental group exhibited higher satisfaction levels compared to the control group. In the experimental group, 50% of patients were somewhat satisfied, 41.7% were undecided, and 8.3% were very satisfied. In contrast, 41.7% of patients in the control group reported being not really satisfied, 33.3% were undecided, and only 16.7% were somewhat satisfied. The chi-square test (χ² = 9.111, p = 0.058) suggests a trend toward higher satisfaction in the experimental group, though the difference was not statistically significant. 

**Table 7:** Patient satisfaction A p-value of p<0.05 was considered statistically significant. Not significant values were denoted as n.s. (n.s.: Not significant)

Satisfaction Level	Experimental Group (n=12) N (%)	Control Group (n=12) N (%)	Chi-Square Test (χ²)	p-value
Not at all satisfied	0 (0%)	1 (8.3%)	9.111	0.058^n.s^
Not really satisfied	0 (0%)	5 (41.7%)		
Undecided	5 (41.7%)	4 (33.3%)		
Somewhat satisfied	6 (50.0%)	2 (16.7%)		
Very much satisfied	1 (8.3%)	0 (0%)		

## Discussion

The EMP intervention has been demonstrated as an effective approach for promoting early recovery in patients undergoing open abdominal surgeries. Previous studies have shown the benefits of EMP in gastrointestinal and cardiac surgeries, further supporting its significance in postoperative care [11]. A study analyzing SICU patients reported an average age of 60 years, ranging from 26 to 81 years, indicating a broad age distribution among SICU admissions. The findings of this study align with this observation, with participants ranging from 31 to 60 years who underwent open cholecystectomy [11].

Research on ICU-managed patients has found a male predominance, with 67.8% of ICU admissions being male [12]. This pattern is consistent with the findings of the present study. Additionally, educational background plays a role in patient demographics, as reported in a study where 57% of surgical ICU patients did not have a high school education, similar to the varying educational levels observed in this study [13].

The effectiveness of early mobilization in ICU patients has been well documented, with studies demonstrating that structured mobility interventions significantly improve physical function and mobility post-ICU. These findings are consistent with the current study, where the experimental group exhibited a substantial increase in mean mobility scores from 1.33 to 6.00, with a mean difference of 4.67 (t = 20.765, p = 0.0001), confirming a highly significant improvement [14].

Additionally, research has shown that rehabilitation protocols in ICU settings improve mobility and reduce the incidence of ICU-acquired weakness, a finding that parallels the results observed in the experimental group of this study [15]. Castro-Avila et al. (2020) demonstrated that early mobilization leads to higher mobility scores and faster recovery from critical illness, further supporting the positive impact of structured mobilization programs on ICU patients [16]. Similarly, Ho et al. (2022) reported significant improvements in functional independence among patients who participated in early mobilization protocols, consistent with the post-test scores observed in the experimental group [15].

Early mobilization within the first few days of ICU admission has been shown to decrease the length of hospital stay, reduce mortality, and improve functionality in mechanically ventilated patients. These findings align with the present study, where the significant chi-square result (p = 0.0001) confirms the effectiveness of the intervention in improving mobility in the experimental group [14,15,17]. Overall, the results emphasize the importance of integrating structured early mobilization protocols into postoperative care to enhance patient recovery outcomes.

Limitations

The study had certain limitations that should be acknowledged. The small sample size may limit the generalizability of the findings, and the short follow-up period restricted the ability to assess long-term outcomes of early mobility interventions. Additionally, while the control group received standard postoperative care, potential variations in individual patient responses could have influenced the results. Future studies with larger sample sizes, extended follow-up periods, and multi-center participation are recommended to further validate these findings and enhance their applicability in clinical settings.

## Conclusions

The implementation of an EMP significantly enhances mobility, functional independence, and overall recovery in patients undergoing open cholecystectomy. The findings demonstrate that early mobilization is effective irrespective of demographic factors, highlighting its broad applicability in postoperative care. Integrating early mobility into standard care practices can reduce recovery time, decrease complications, and improve patient satisfaction. The strong correlation between mobility and functional independence underscores the protocol’s impact on rehabilitation.
